# Supplementary figures and images for: Prolonged development of long-term potentiation at lateral entorhinal cortex synapses onto adult-born neurons
Source: PLoS One. 2021 Jun 18;16(6):e0253642. doi: 10.1371/journal.pone.0253642 (PMC8213073; doi:10.1371/journal.pone.0253642)

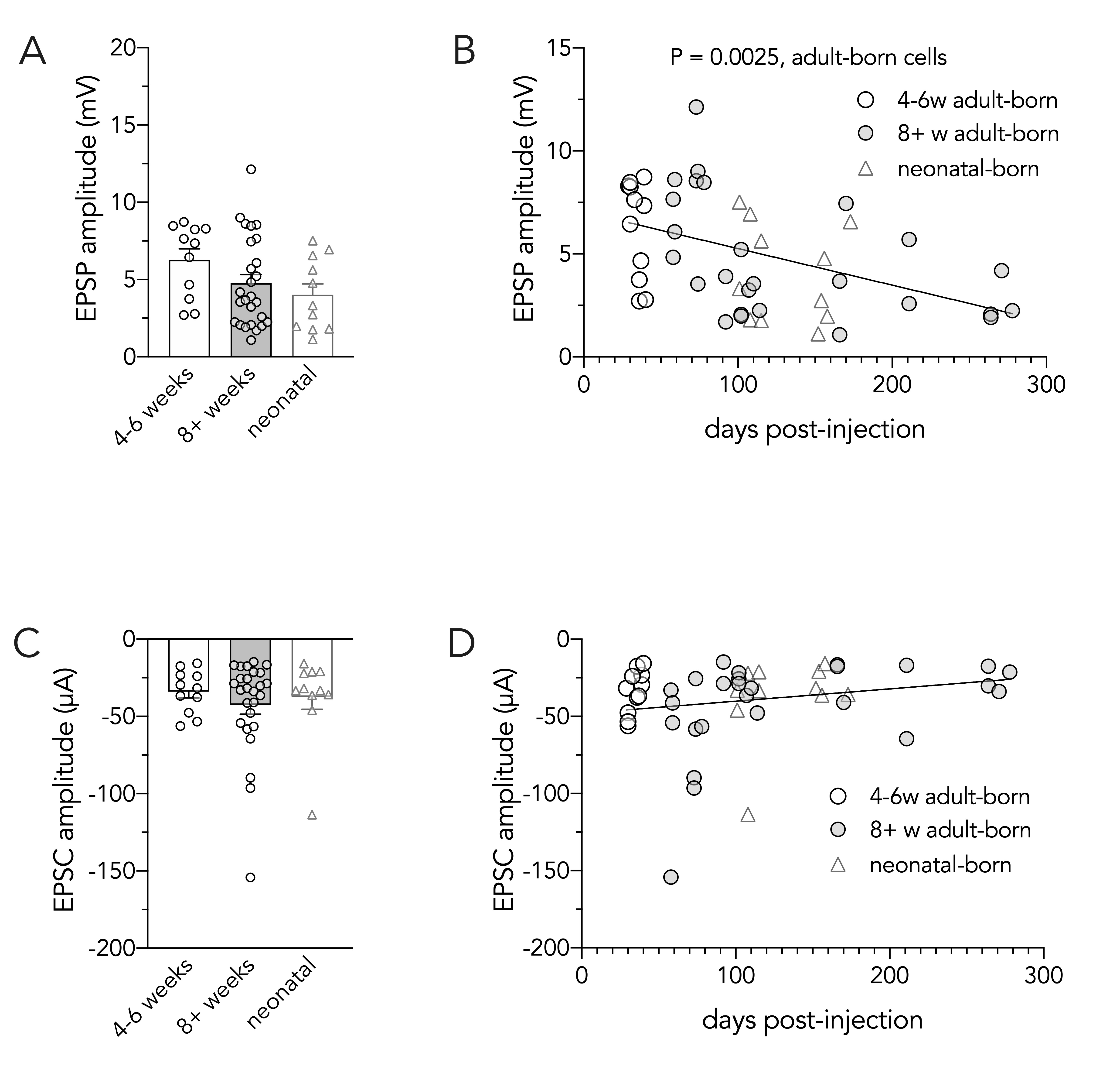

Supplement: S1 Fig — A) Baseline peak EPSP amplitudes did not significantly differ across groups (Kruskal Wallis test, P = 0.09). Among adult-born cells, EPSP amplitude negatively correlated with days post-tamoxifen injection (R2 = 0.23, P = 0.0025). C) Among adult-born cells, EPSC amplitude did not vary across groups (Kruskal Wallis test, P = 0.9). D) EPSC amplitude did not correlate with days post-tamoxifen injection (R2 = 0.05, P = 0.18). Bars reflect mean ± standard error. (JPG) [file pone.0253642.s001.jpg]
